# Proteogenomics Reveals Microproteins in Activated T Cells

**DOI:** 10.1016/j.mcpro.2025.100914

**Published:** 2025-02-04

**Authors:** Yang Yang, Chuangmiao Chen, Kecheng Li, Yuanliang Zhang, Lei Chen, Jue Shi, Quanhua Mu, Yang Xu, Qian Zhao

**Affiliations:** 1 State Key Laboratory of Chemical Biology and Drug Discovery, Department of Applied Biology and Chemical Technology, The Hong Kong Polytechnic University, Hong Kong, China; 2 Centre for Eye and Vision Research, 17W Hong Kong Science Park, Hong Kong, China; 3 School of Medicine, Southern University of Science and Technology, Shenzhen, China; 4 Laboratory for Synthetic Chemistry and Chemical Biology Limited, Hong Kong, China; 5 Center and Quantitative Systems Biology, Department of Physics, Hong Kong Baptist University, Hong Kong, China

**Keywords:** proteogenomic profiling, microproteins, small ORFs, T cell activation, quantitative proteomics

## Abstract

Noncanonical micropeptides, or called novel microproteins, *i.e.*, polypeptides mostly under 10 kDa, are encoded by genomic sequences that have been previously annotated as noncoding but now known as small open reading frames (sORFs). The recent identification of microproteins encoded by sORFs has provided evidence that many sORFs encode functional microproteins that play crucial roles in various biological processes. T cell activation is a critical biological process for adaptive immune response. Understanding key players in this process will allow us to decipher the complex mechanisms as well as develop immunotherapy for treating a wide range of diseases. Although there have been extensive studies on canonical proteins in T cell activation, the novel microproteins in T cells and their roles have been uncharted water to date. Nascent proteins are defined as newly synthesized polypeptides that emerged during the translation of mRNA. In this study, we combined nascent proteomics and quantitative proteomics to identify 411 novel microproteins in primary human T cells, including 83 nascent microproteins. We activated the T cell function with either PMA/Ionomycin (distal activation) or CD3/CD28 activating antibodies (proximal activation) and obtained a comprehensive canonical protein and microprotein profiles to pinpoint common and distinct differentially expressed proteins under these two activation conditions. After experimental testing, three microproteins numbered T1, T2 and T3 were found to be functional in regulating T cell activation. Bioinformatic and proteomic analyses suggested that T1 was functional related to immune as negative feedback to T cell activation. Our study not only established an integrated approach to uncover and elucidate novel microproteins but also highlights the significant role of microproteins in regulating T cell activation.

## Highlights

• 83 nascent and 328 pre-existing microproteins were identified in human T cells.
• Canonical protein and microprotein profiles were compared in 2 activation ways.
• Microproteins were found to function as novel suppressors in T cell activation feedback.

## Graphical Abstract

**Figure:**
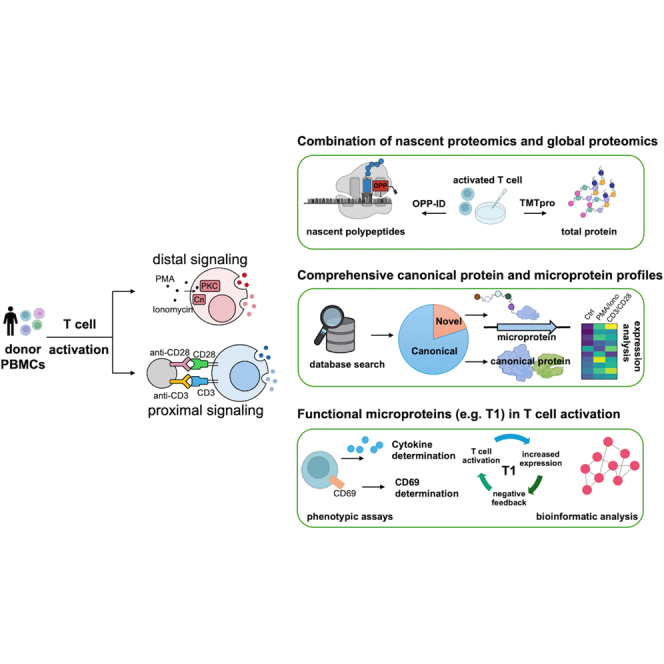

## In Brief

In this study, we have combined nascent proteomics and quantitative proteomics to identify 411 novel microproteins in primary human T cells and quantify the changes in microproteins under proximal and distal signal activation. Several microproteins were found to be functional in regulating T cell activation with cytokine production assays. Further mechanism studies showed that a representative microprotein named T1 contributed to negative feedback to suppress drastic activation of T cells. Our findings not only facilitate the unveiling of novel microproteins with an integrated approach but also highlight the significance of microproteins that may play a critical role during T cell activation.

Noncanonical microproteins are encoded by unannotated DNA sequences that are known as noncoding sequences. The discovery of microproteins encoded by sORFs has challenged our conventional perception of the genome as being predominantly composed of protein-coding genes and non-coding regions (1, 2). The recent identification of microproteins encoded by sORFs has provided evidence that many sORFs actually encode functional microproteins that play crucial roles in various biological processes such as gene regulation (3, 4, 5), signal transduction (4, 6), and stress response (7, 8). The discovery of these microproteins will not only improve our understanding of the human genome and proteome but also will provide insights into the mechanisms that generate functional diversity and the evolution of the genome or may even offer new therapeutic targets for disease treatments. However, this part of the human proteome remains largely underexplored to date.

T cell activation is a pivotal process in the adaptive immune response, enabling T cells to recognize and respond to specific antigens effectively (9). This activation is crucial for the proliferation and differentiation of T cells, ultimately facilitating robust immune responses against pathogens and tumor cells (10). There are two widely utilized methods for activating T cells (11, 12). Distal activation uses phorbol myristate acetate (PMA) in conjunction with ionomycin (Iono) while proximal activation engages CD3 and CD28 co-stimulation. PMA/Iono activates protein kinase C (PKC) and elevates intracellular calcium levels, resulting in immediate and broad activation independent of antigen specificity (13, 14). CD3/CD28 activates the T cell receptor (TCR) through CD3, requiring specific antigen recognition and co-stimulatory signals from CD28, thereby promoting a more regulated and physiological response (11, 15, 16). Although canonical proteins that are involved in T cell activation have been reported, there is limited study to directly compare distal and proximal activation pathways. Moreover, the microprotein players in this important cellular process have not been discovered to date.

When cells encounter stress or external stimuli, dynamic alterations in the translatome can be triggered (17, 18). These newly synthesized proteins serve as critical indicators of cellular responses, offering valuable insights into the underlying biochemical pathways activated during such events (19, 20). We have previously developed a method O-propargyl puromycin (OPP)-ID to label and enrich newly synthesized proteins for downstream mass spectrometry (MS) analysis (21).

To identify microproteins that are involved in T cell activation, we combined the chemical proteomic method OPP-ID and TMTpro-based global proteomics to depict the landscape of microproteins in human unstimulated and activated T cells. RNA-seq was performed with CD3+ T cells isolated from human peripheral blood mononuclear cells (PBMCs) for microprotein database construction. In total 411 microproteins were identified, including 83 nascent microproteins and 328 pre-existing microproteins. Among them, three microproteins numbered T1, T2, and T3 were found to be functional in regulating T cell activation.

## Experimental Procedures

### Chemicals and Reagents

Acetonitrile, methanol, and formic acid were from Thermo Fisher Scientific. Lysyl endopeptidase (mass spectrometry grade) and trypsin (sequencing grade) were purchased from Promega. Ionomycin (Iono) was purchased from STEMCELL Technologies. O-Propargyl-Puromycin was obtained from MedChemExpress. Ficoll, dithiothreitol (DTT), iodoacetamide (IAM), phorbol 12-myristate 13-acetate (PMA), and all other reagents were from Sigma Aldrich.

### Primary T Cell Isolation and Activation

Human primary T cells were isolated from peripheral blood mononuclear cells (PBMC) with a negative selection method using the EasySep Human T Cell Enrichment Kit (STEMCELL Technologies, #19051), according to the manufacturer's protocol. PBMCs were separated from fresh human buffy coats of healthy donors (blood samples from the Hong Kong Red Cross) by Ficoll solution (Cytiva, #17144003). Informed written consent from the blood donors was obtained by the Hong Kong Red Cross in accordance with its ethical guidelines. T cell activation was performed either with PMA 25 ng/ml and Iono 1 μg/ml or with anti-CD3/CD28-coated microbeads (ThermoFisher Scientific, #11161D) at a bead-to-cell ratio of 1: 1, at 37 °C with 5% CO_2_ for 24 h.

### Detection of Interferon-Gamma (IFN-γ)

Detection of IFN-γ by enzyme-linked immunospot (ELISpot) was performed according to the manufacturer’s protocol using the ELISpot PLUS ALP kit (Mabtech). 2.0 × 10^5^ human primary T cells or Jurkat T cells cells/well were seeded in the 96-well plate and stimulated by PMA/Iono or CD3/CD28 for 24 h at 37 °C with 5% CO_2_. Spots were pictured using a stereomicroscope and counted by Image J (Version 1.51).

### Detection of Interleukin-2 (IL-2)

Human primary T cells or Jurkat T cells were plated at 2.0 × 10^5^ cells/well with RPMI-1640 medium before being incubated with the PMA/Iono or CD3/CD28 stimuli for 24 h. The supernatant was then aliquoted for IL-2 measurement using human IL-2 ELISA kit (ThermoFisher Scientific, #BMS221-2) following the manufacturer’s protocol. Absorbance was measured at 370 nm using a microplate reader.

### RNA-seq Analysis

Primary T cells were harvested and washed with ice-cold PBS buffer twice. Total RNA was extracted with a TRIzol reagent (Sigma, #T9424). Quality assessment of total RNA was performed by Agilent 2100 Bioanalyzer (Agilent Technologies). RNA samples with RNA integrity number (RIN) bigger than 9 were subjected to RNA sequencing. All the RNA samples were transferred to Novogene for library construction and sequencing. Briefly, mRNA was enriched from total RNA using poly-T oligo-attached magnetic beads. After fragmentation, the first strand cDNA was synthesized using random hexamer primers followed by the second strand cDNA synthesis. The library was ready after end repair, A-tailing, adapter ligation, size selection, amplification, and purification. Libraries that passed the quality control procedure were sequenced on Hiseq 4000 (Illumina). Around 100 M PE150 reads were generated from each sample.

### Retrovirus Production and Transduction of Jurkat Cells

To produce the retroviral supernatant, HEK293T cells were cotransfected with retroviral vectors, Peg-Pam-e plasmid, and the DRF plasmid, using the Mirius TransIT-LT1 transfection reagent (Mirus). The supernatant containing the retrovirus was collected 48 and 72 h later. For transduction, 0.25 × 10^6^ Jurkat cells were transduced with retroviral supernatants using retronectin (Takara)- precoated Non-Tissue Culture Treated Plate 24-well (Falcon). On Day 3, transduced Jurkat cells were collected from retronectin-coated plates and expanded in a fresh complete RPMI medium. The stable cell lines were screened with 5 μg/ml and 10 μg/ml puro for 24 h to complete the selection.

### CD69 Determination by Flow Cytometry

For cell-surface staining, cells were incubated with antibodies at room temperature for 15 min or at 4 °C for 30 min. FITC anti-human CD69 Antibody (clone FN50) was obtained from BioLegend. All flow cytometric data were analyzed using FlowJo (BD Biosciences, v.10.6.2).

### Nascent Protein Profiling, LC-MS/MS

1.0 × 10^7^ T cells with or without 24 h activation were divided into five conditions for treatments: 1) PMA/Iono-activated cells treated with cycloheximide (CHX) and OPP (defined as “PMA/Iono+CHX+OPP”) as a control for reduced translation; 2) CD3/CD28-activated cells treated with CHX and OPP (defined as “CD3/CD28+CHX+OPP”) as a control for reduced translation; 3) unstimulated cells treated with OPP (defined as “OPP”) as a control for activation; 4) PMA/Iono-activated cells treated with OPP (defined as “PMA/Iono+OPP”); 5) CD3/CD28-activated cells treated with OPP (defined as “CD3/CD28+OPP”). CHX (50 μg/ml) was added to cells 30 min before the addition of OPP (30 μM). After treated with OPP for another 30 min, cells were collected and lysed in lysis buffer containing 100 mM HEPES, PH 7.5, 150 mM NaCl, 1% NP-40, 2 mM PMSF, 1× protease inhibitor and 0.1% benzonase. OPP-labeled proteome was conjugated to biotin-azide with click chemistry according to our previous report (21). Biotin–polypeptide complexes were purified by streptavidin beads for on-bead digestion and LC-MS/MS analysis. LC-MS analyses were performed on a Orbitrap Exploris 480 Mass Spectrometer (Thermo Fisher Scientific) coupled with UltiMate 3000 nano UPLC system (Thermo Fisher Scientific). Peptides were injected into LC and separated by a 2-h gradient on the 25 cm C18 column (IonOpticks, Australia). Data-independent acquisition (DIA) was set as data acquisition mode. MS1 was set at 60,000 and the precursor range started from 400 to 1000. The window was 10 *m/z*. MS2 resolution was set as 30,000 at 32% HCD collision energy.

### Global Proteomics, LC-MS/MS

5 × 10^6^ primary T cells with or without 24 h activation was lysed in lysis buffer containing 100 mM HEPES, PH 7.5, 150 mM NaCl, 1% NP-40, 2 mM PMSF, 1× protease inhibitor and 0.1% benzonase. Proteins were then reduced with 10 mM dithiothreitol (DTT) and incubated at 45 °C for 30 min, and further alkylated with 30 mM iodoacetamide (IAM) at RT for 30 min in the dark. Lysates were digested by MS-grade Lys-C (Wako) at an enzyme/protein ratio of 1:200 (w/w) for 2 h at 37 °C. A secondary digestion was performed by adding sequencing-grade trypsin (Promega) at an enzyme/protein ratio of 1:50 (w/w) for additional 12 h at 37 °C. The tandem mass tags (TMT) labeling was performed according to the manufacturer with a TMT/peptide ratio of 10:1 (w/w) (TMTpro-16 plex, Thermo Fisher Scientific). TMTpro-labeled peptides were fractionated through high pH reverse phase column into 10 fractions prior to LC-MS/MS analysis. LC-MS/MS analyses of retina samples was performed on an Orbitrap Exploris 480 mass spectrometer (Thermo Fisher Scientific) coupled with an UltiMate 3000 UPLC system (Thermo Fisher Scientific). A RSLC C18 analytical column (75 μm × 250 mm, 2.0 μm, 100 Å) (Thermo Fisher Scientific) was employed for LC separation. Data were collected in data-dependent acquisition (DDA) mode. The top 10 precursor ions with a charge state of 2+ or higher were fragmented by HCD. The MS1 Orbitrap resolution was set at 60,000 and the MS1 AGC target was set at 4 × 10^5^. The MS2 Orbitrap resolution was set at 30,000 while the MS2 AGC target and the maximum injection time were set at 1 × 10^5^ and 54 ms.

### Data Analysis and Processing

For microprotein database, we combined databases from three sources: (1) public-available downloaded databases, including OpenProt (https://www.openprot.org/, version number 1.6) containing 557,568 entries, sORFs.org (http://www.sorfs.org) containing 601,636 entries, and SmProt (http://bigdata.ibp.ac.cn/SmProt/) containing 393,285 entries; (2) RNA-seq-based database containing 507,195 entries; (3) Ribo-seq-based database containing 409,024 entries. For canonical protein database, we downloaded protein sequences from Swiss-Prot (Release June 2020, 20,368 human entries).

For DIA data analysis (“The sORF-Encoded Microproteins in T Cells and Differentially Expressed during T Cell Activation” and “Signaling Pathways Related to the Regulatory Function of a Microprotein T1”), DIA-NN (version 1.8.1) was used to create the spectral library from the combined databases. The ∗.speclib file was generated using the default parameters of DIA-NN, which included a 1% false discovery rate (FDR). The software parameters were set as follows: Peptide length range from 7 to 30, precursor charge range from 1 to 4, precursor *m/z* range comprised between 300 and 1800, fragment ion *m/z* range between 200 and 1800, 1% precursor FDR. After obtaining the library, DIA-NN was used to analyze DIA data with the Match-between-run (MBR) search mode to reduce the library's redundancy. The search parameters were consistent with those used in the establishment of the spectral library. Carbamidomethylating on cysteine was set as a fixed modification, and oxidation of methionine residues and acetyl protein N-term were set as variable modifications. One missed cleavage and trypsin protease mode was selected.

For TMTpro-DDA data analysis (“Overall Proteome Landscape of T-Cell Activation Triggered by Proximal and Distal Signals”), TMTpro data were searched by the SEQUEST algorithm (Proteome Discoverer 2.5, Thermo Fisher Scientific). TMTpro tags on lysine residues and peptide N terminal, as well as carbamidomethylation on cysteine, were set as fixed modifications, oxidation of methionine residues and acetyl protein N-term were set as variable modifications. TMTpro labeling efficiency was determined using global samples with labeling set as a variable modification. Precursor ion mass tolerance and fragmentation tolerance were set as 10 ppm and 0.02 Da for the database search. The peptide length range was set from 6 to 144 as default. Trypsin was set as the enzyme with max. Missed cleavage sites two. A workflow of consensus node ‘CWF_Comprehensive_Enhanced Annotation_Reporter_Quan_forTMTpro’ was applied, and the main parameters were set as follows: Reported FASTA title lines with best match mode, confidence thresholds of peptide FDR and protein FDR were set as 0.01, peptide validator of target/decoy selection for PSM level FDR calculation with automatic mode, protein grouping with strict parsimony principle, co-isolation threshold: 50, average reporter S/N threshold: 10, and minimum PSM confidence: high.

Canonical proteins with at least two unique peptides were used for quantification. For microproteins, one unique peptide was used for quantification. The average values of intensities were used for the calculation of fold changes after the Log2 transformation. After data searching of microproteins, sequential mapping, and BLAST algorithm were applied to remove canonical proteins. First, peptide sequences that can be found in any reference proteins in the library (Refprot, downloaded from OpenProt 1.6, 141,395 human entries) were removed. Secondly, the detected peptide sequences were further BLAST against Swiss-Prot (Release June 2020, 20,368 human entries). A cut-off of 80% in sequence similarity was applied to exclude any sequence that is similar to known protein in Swiss-Prot.

### Experimental Design and Statistical Rationale

For the nascent proteome (OPP-ID) in “The sORF-Encoded Microproteins in T Cells and Differentially Expressed During T Cell Activation”, we used primary T cells for nascent protein enrichment in three doners, including three conditions unstimulated, stimulated by PMA/Iono, and stimulated by anti-CD3/CD28. We included technical (n = 2) and biological replicates (n = 3 from 3 donors) to average out variability. The data were continuously collected by MS in the same batch. For the TMTpro-global proteome study in “Overall Proteome Landscape of T-Cell Activation Triggered by Proximal and Distal Signals”, we used primary T cells in three biological replicates from three doners, including three conditions unstimulated, stimulated by PMA/Iono, and stimulated by anti-CD3/CD28 (n = 3). The three samples within the same condition were discontinuous and randomized in TMTpro labeling. For the RNA-seq in Overall Proteome Landscape of T-Cell Activation Triggered by Proximal and Distal Signals, we used primary T cells in three biological replicates from three doners, including three conditions unstimulated, stimulated by PMA/Iono, and stimulated by anti-CD3/CD28 (n = 3). For the global proteome study in “Signaling Pathways Related to the Regulatory Function of a Microprotein T1”, we used Jurkat cells in three biological replicates (n = 3), including three conditions: unstimulated in mock group (Jurkat cells transfected with empty vector), stimulated by anti-CD3/CD28 in mock group, and stimulated by anti-CD3/CD28 in the gene-overexpressed group (Jurkat cells transfected with T1). Benjamini-Hochberg adjusted *p* values were used for both RNA-seq data and proteomics data analyses and *p* < 0.05 was the statistical limit of significance. In the ELISpot assays, we used primary T cells in 3.2 section and Jurkat cells in 3.4 section (n = 3). In the flow cytometry assay, we used Jurkat cells in 3.5 section (n = 5). Data were analyzed by a two-tailed unpaired Student's *t* test, and *p* < 0.05 was the statistical limit of significance. ∗ and ∗∗ indicated *p* < 0.05 and *p* < 0.01, respectively. Unless otherwise stated, all the data in the graphs were expressed as arithmetic mean ± the SD.

## Results

### Strategies to Discover Microproteins in T Cells

**Fig. 1:**
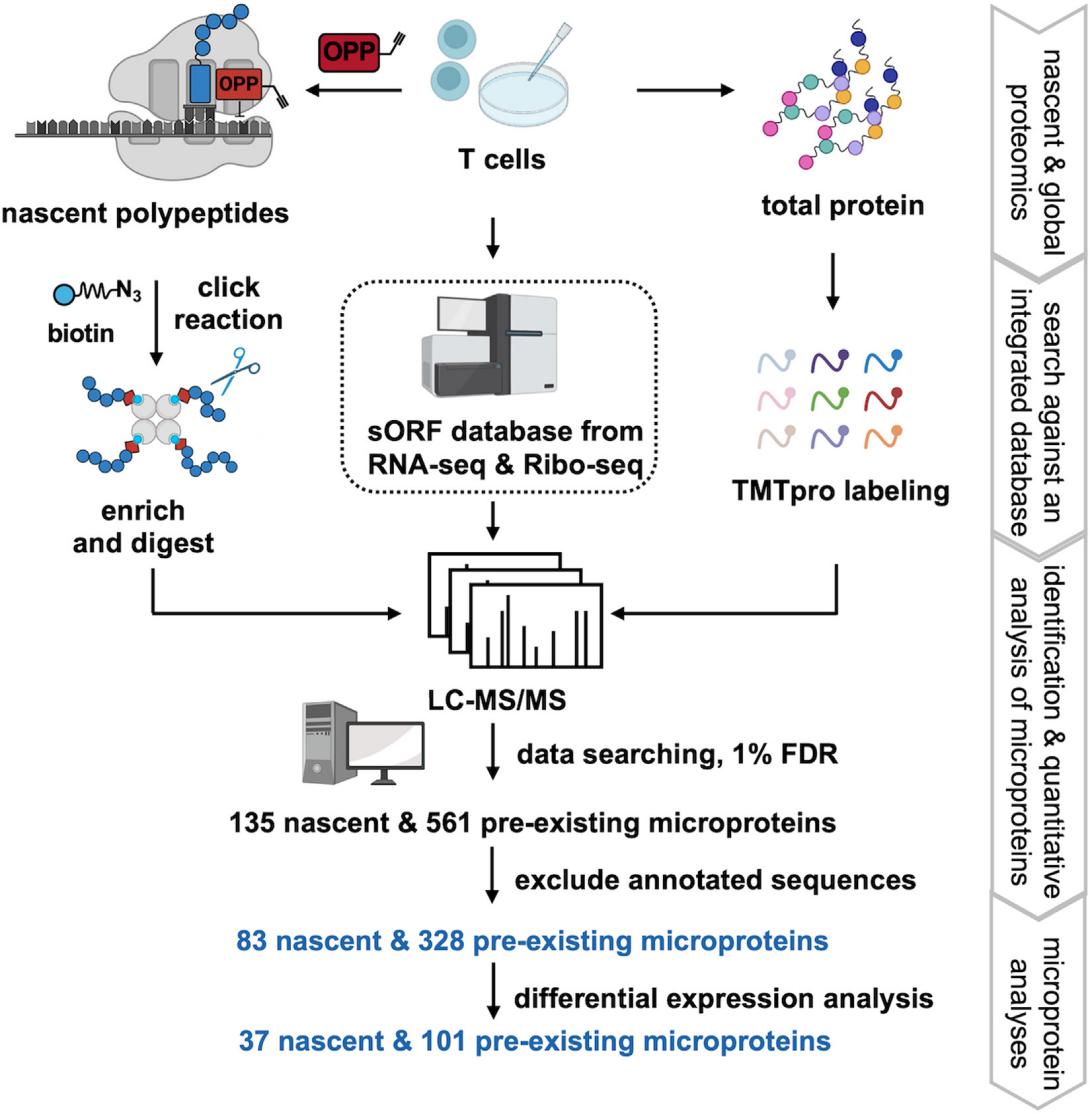
**The workflow for MS-based discovery of nascent and pre-existing microproteins in activated human T cells**. The workflow consists of CD3+ T cell isolation and activation by PMA/Iono and anti-CD3/CD28, sORF database construction using RNA-seq and Ribo-seq data, O-propargyl-puromycin (OPP)-based nascent protein pull-down approach, and TMTpro-based quantitative proteomics approach.

We designed an integrated workflow to achieve a comprehensive identification of microproteins from T cells (Fig. 1). The workflow consists of three steps: (a) An OPP-ID strategy was utilized to enrich nascent polypeptides by metabolically incorporating OPP into the C terminus of nascent polypeptides during elongation. As OPP carries a terminal alkyne group as a handle, the OPP-labeled proteome was conjugated to biotin-azide *via* click chemistry, and subsequently captured with streptavidin beads, followed by digestion and MS analysis based on our optimized DIA strategy (22). (b) In parallel, the total protein extract was harvested from unstimulated and stimulated T cells and labeled with TMTpro for quantitative proteomics analysis at a global proteome scale. (c) RNA sequencing (seq) was conducted to depict transcriptome changes and to construct a sample-specific sORF database through three-frame translation. Then MS data for nascent proteomics and global proteomics analyses was searched against an integrated sORF database composed of the sample-specific database and public database based on Ribo-seq data and the other available databases. To distinguish novel microproteins from canonical proteins or protein fragments, we applied two stringent filtering criteria. First, peptide sequences that can be found in any reference proteins (Refprot, downloaded from OpenProt 1.6, 141,395 entries) were removed. Secondly, even if it is unique, the peptide sequences of microproteins still must show similarity no more than 80% with any known protein (Swiss-Prot, Release June 2020, 203,68 human entries), so that isoforms or mutation can be excluded. By employing this workflow, we have identified 83 nascent and 328 pre-existing microproteins in T cells with one unique peptide and quantified them to find the differentially expressed ones with statistical significance. The confidence of identification was evaluated by comparing each spectrum with theoretical one. The closer the similarity score approaches 1.0, the greater the similarity between the experimental spectrum and the theoretical spectrum. The majority (87.4%) of identifications in the nascent microprotein profiling with a similarity score exceeding 0.5 indicate reliable identifications. Furthermore, 67.0% identifications present a similar score greater than 0.7, demonstrating high confidence (Supplemental Fig. S1).

### Overall Proteome Landscape of T-Cell Activation Triggered by Proximal and Distal Signals

**Fig. 2:**
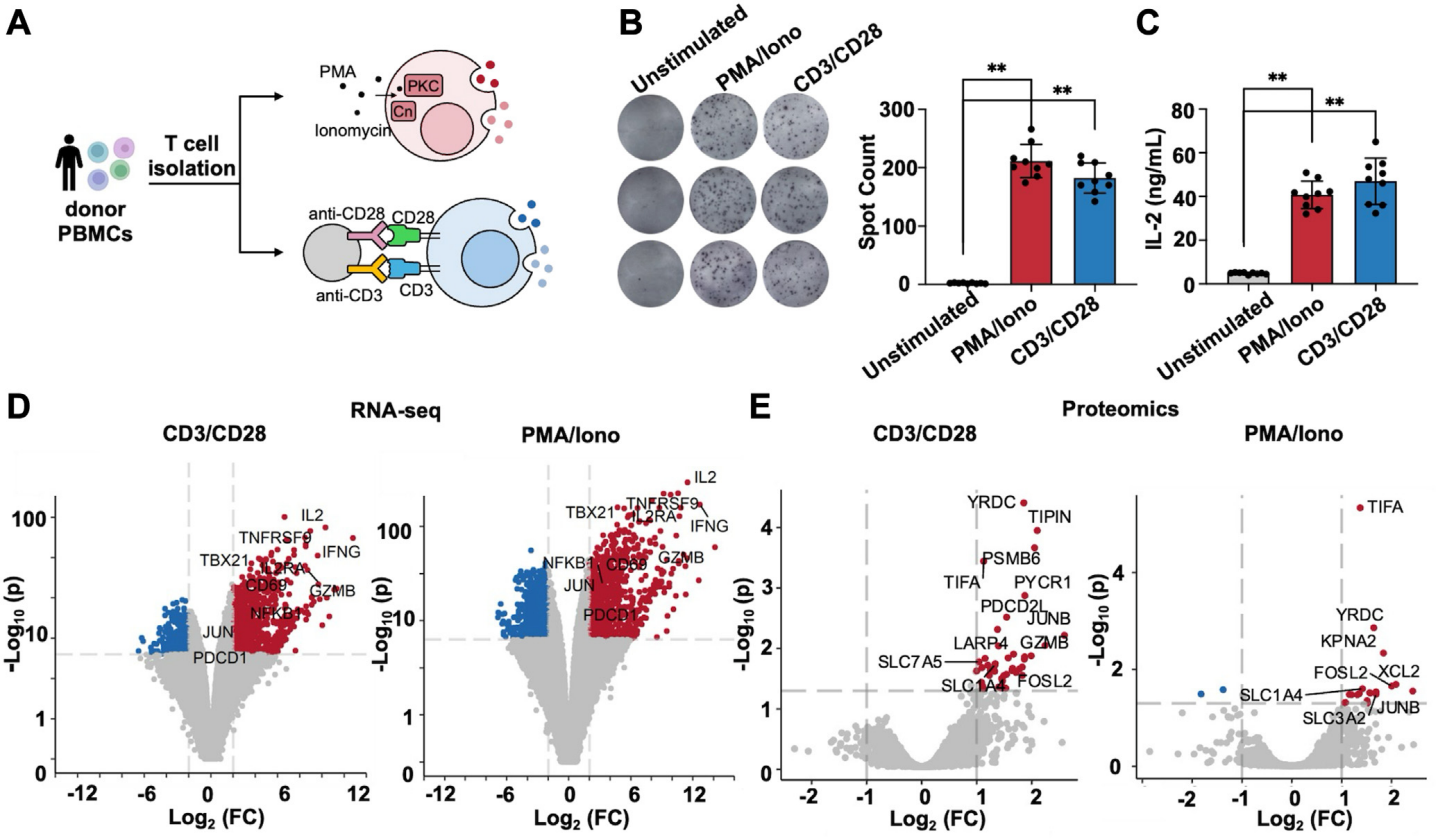
**The overall proteome landscape of T-cell activation under two different stimuli**. *A*, illustration of T cell activation *in vitro* by the two method, PMA/Iono and anti-CD3/CD28 antibodies. *B*, cytokine production in the primary T cell after activation. IFN-γ secretion was assessed using ELISpot assays. *Left*, representative results of the primary T cells from different donors are listed. Experiments were conducted in triplicates. *Right*, data is presented as the mean ± s.d. ∗∗*p* < 0.01 by Student’s *t* test. *C*, IL-2 production assessed using ELISA. Experiments were conducted in triplicates. Data is presented as the mean ± s.d. ∗∗*p* < 0.01 by Student’s *t* test. PMA/Iono, primary T cells were activated by PMA/ionomycin. CD3/CD28, primary T cells were activated by anti-CD3/CD28. *D*, Volcano plots of the canonical proteins during T cell activation which were identified in RNA-seq data. *Red*, genes with fold change >4 and adjusted *p* value < 0.01; *blue*, gene with fold change <0.25 and adjusted *p* value < 0.01. *E*, Volcano plots of the canonical proteins during T cell activation which were identified in proteomics data. *Red*, proteins with fold change >2 and adjusted *p* value < 0.05; *blue*, proteins with fold change <0.5 and adjusted *p* value < 0.05. The calculation of adjusted *p* values was performed by Benjamini-Hochberg procedure.

T cells were isolated from human PBMCs from three healthy human donors and activated *in vitro* with either PMA/Iono or anti-CD3/CD28 agonistic antibodies (Fig. 2*A*). PMA stimulates PKC and Iono increases intracellular calcium levels, resulting in activation of NFAT (23). On the other hand, anti-CD3/CD28 agonistic antibodies work through ZAP-70, which phosphorylate LAT and SLP-76 to activate T cells (23). In our experiment, both methods effectively induced T cell activation, as evidenced by over 90-fold enhancement in the IFN-γ secretion and 8-fold increase in IL-2 production (Fig. 2, *B* and *C*). Next, we conducted RNA-seq and global proteomics analyses to identify and compare the differentially expressed genes (DEGs) and proteins (DEPs) associated with each activation method (Fig. 2, *D* and *E*). In RNA-seq, 1019 genes were up-regulated (fold change >4 and adj. *p* value < 0.01) and 1020 genes were down-regulated (fold change <0.25 and adj. *p* value < 0.01) genes when T cells were activated by PMA/Iono. When T cells were treated with anti-CD3/CD28 antibodies, there were 854 up-regulated and 490 down-regulated genes according to RNA-seq results. Among these, 37% (870 genes) of the identified DEGs were consistent with previously reported gene expression profiles during human T-cell activation (24, 25). Both activation methods resulted in the upregulation of 1043 common DEGs (Supplemental Fig. S2), including IL2, TNFRSF9, IFNG, IL2RA, TBX21, GZMB, CD69, NFKB1, JUN, and PDCD1 (26, 27, 28, 29, 30), etc. With proteomic analysis, we identified 19 up-regulated (fold change >2 and adj. *p* value < 0.05) and 2 down-regulated (fold change <0.5 and adj. *p* value < 0.05) proteins during activation by PMA/Iono, and 46 up-regulated (fold change >2 and adj. *p* value < 0.05) genes in CD3/CD28 group (Supplemental Table S1). Among all the identified DEPs, 44% were detected in both groups (Supplemental Fig. S1), including SLC1A4, SLC3A2, TIPIN, YRDC, JUNB, TIFA, and FOSL2, etc. For example, the amino acid transporters that are important for glutamine uptake (SLC1A4 and SLC3A2) were upregulated upon stimulation, to support the metabolic demands of activated T cells for amplified protein synthesis and accelerated proliferation (31). We also observed cytokine expression specifically induced by PMA/Iono, such as XCL2, PREPL, PRKCA, and TAGAP, while anti-CD3/CD28 antibodies preferentially induced LARP4, GZMB, and SLC7A5 (32). We also identified a few potential novel markers of T-cell activation from the commonly identified DEPs shared by the two groups, such as FOSL2 which is a component of the AP-1 transcription factor (33). Taken together, our results highlight the similarities and discrepancies between PMA/Iono and CD3/CD28 activation methods and provide insights into the molecular mechanisms underlying T cell activation.

### The sORF-Encoded Microproteins in T Cells and Differentially Expressed during T Cell Activation

**Fig. 3:**
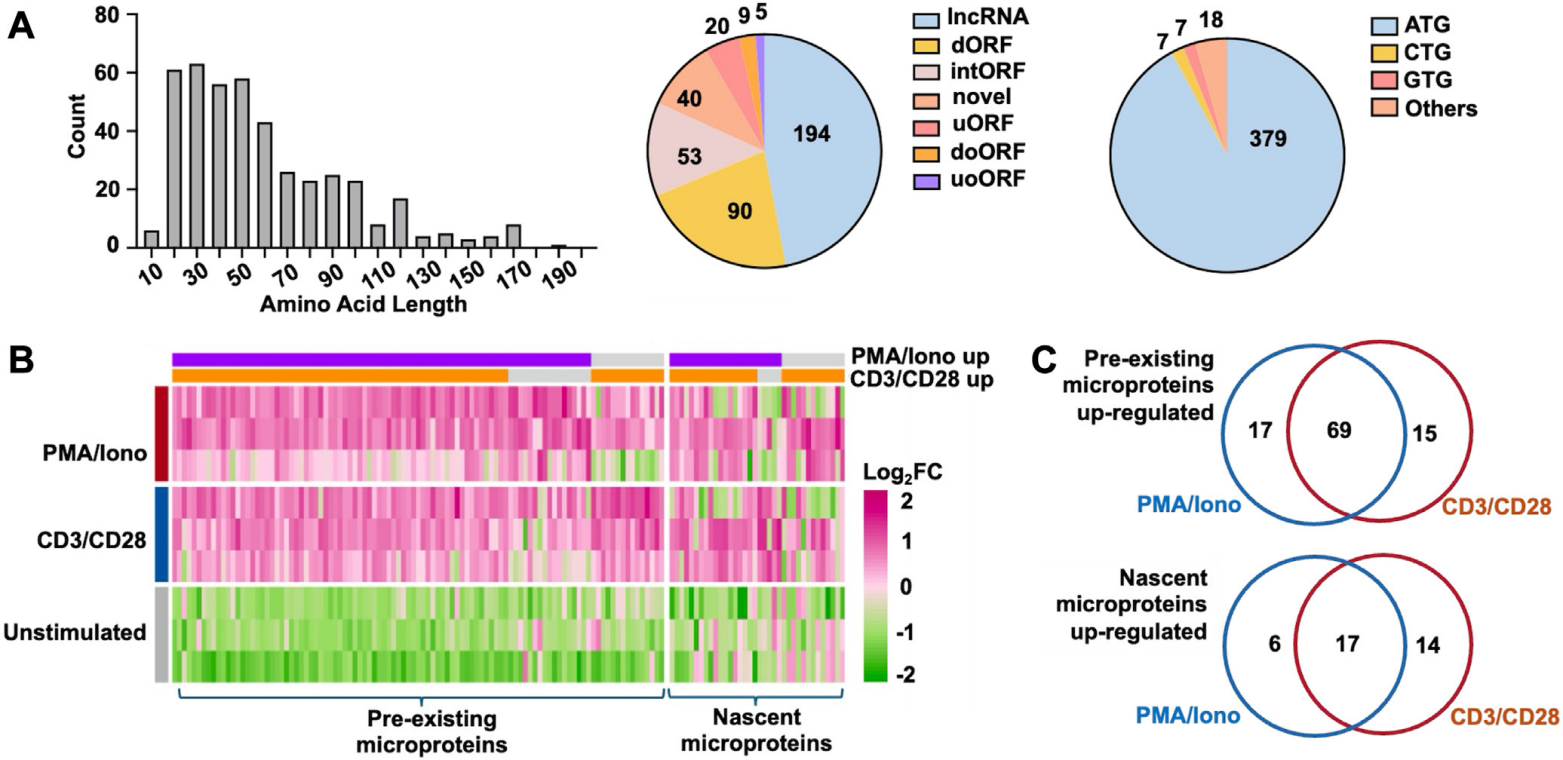
**The sORF-encoded microproteins identified in T cells and differentially expressed in activated T cells**. *A*, characteristics of microproteins. *Left*, length distribution; middle, sORF classification; *right*, start codon distribution. lncRNA, long non-coding RNA; dORF, downstream ORF; intORF, internal ORF; novel, novel ORF; uORF, upstream ORF; doORF, downstream overlapped ORF; uoORF, upstream overlapped ORF. *B*, a heatmap of the microproteins identified with two proteomic methods, under indicated treatment conditions. The *purple* grid indicates the differentially expressed microproteins triggered by PMA/Iono (distal signaling). The *orange* grid indicates the differentially expressed microproteins triggered by CD3/CD28 (proximal signaling). The *gray* grid shows the microproteins that are specifically up-/down-regulated by respective treatment condition. *C*, Venn-diagrams of up-regulated microproteins identified in the two activation groups.

Further analysis of the 411 newly identified microproteins showed interesting characteristics. As expected, most microproteins are less than 100 amino acids in length, with a median value of 44. The rest microproteins between 100 to 200 amino acids took 12.0% (Fig. 3*A* left). Based on the recently proposed classification and nomenclatures (34), the sORFs encoding microproteins from T cells were classified into seven categories. The majority microproteins, taking 47%, were encoded by lncRNAs, while about 27% were encoded by the upstream ORF (uORF) and downstream ORF (dORFs) of a canonical gene. A considerable percentage of novel microproteins were translated from frameshift sequences within a canonical gene, which was named “intORF” in this study. For the 40 microproteins derived from novel transcripts that aren’t documented in Genebank or Ensembl database, the sORFs were named “novel” (Fig. 3*A* middle). Although most of sORFs use ATG as start codon, there was 7.8% sORF with alternative start codon, such as CTG, GTG, etc. Non-ATG initiation in microprotein translation was repeatedly reported with a mysterious molecular mechanism and this will be our research focus in due course (35) (Fig. 3*A* right). With MS quantification, we were able to identify differentially expressed microproteins under T cell activation under two different signaling mechanisms (Supplemental Table S2). Albeit there was noticeable variance among the individual donors, a principal component analysis (PCA) analysis showed sufficient correlation of biological replicates and differentiation between different treatment groups (Supplemental Fig. S3). We found that the majority, or 61%, of the differentially expressed microproteins were triggered by both distal (purple grid in Fig. 3*B*) and proximal (orange grid in Fig. 3*B*) signaling pathways, similar to that of canonical proteins. There were also microproteins that are specifically up-/down-regulated by respective treatment condition (gray grid in Fig. 3*B*). Such observation was consistent regardless of pre-existing proteins or newly synthesized microproteins identified with different proteomics methods (Fig. 3*C*). There were a few upregulated sORFs that were previously annotated as lncRNA such as ENST00000663478.1 (36) and IP_724881 (37). Interestingly, these lncRNAs were taken granted as noncoding and reported to function at the RNA level, without investigating whether they have translation potential or whether the translated peptides play a role. Our study of microproteins offers a novel perspective on the functions of lncRNAs and identifies new targets for future studies.

### Novel Microproteins Regulate T Cell Activation

**Fig. 4:**
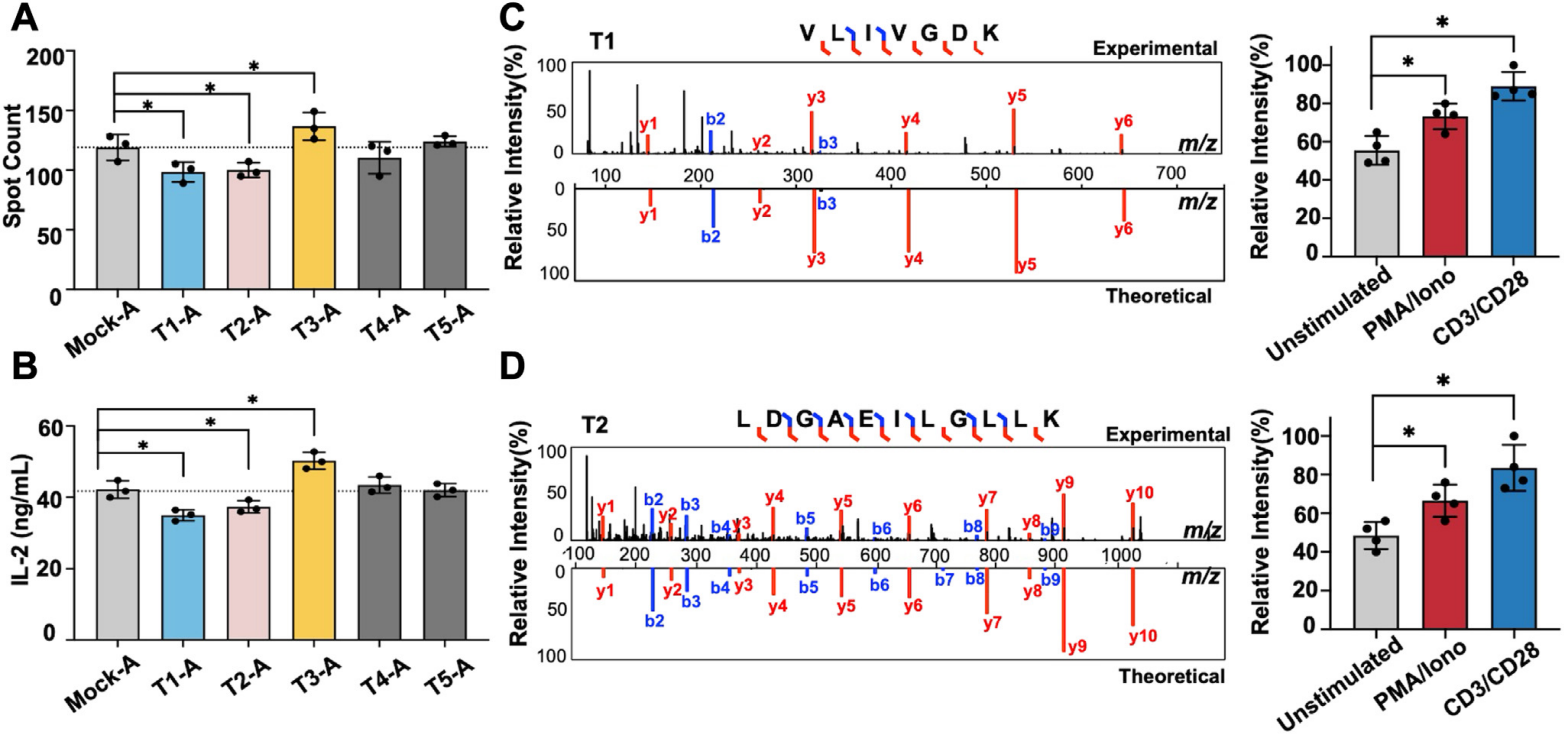
**Examination of microproteins’ functional roles in T cell activation**. *A*, IFN-γ secretion was assessed for the selected microproteins using ELISpot assays. Jurkat cells overexpressing the microproteins or empty vector (Mock) were activated using anti-CD3/CD28 antibodies. Experiments were conducted in triplicates. Data is presented as the mean ± s.d. ∗*p* < 0.05 by Student’s *t* test. A: activated by CD3/CD28. *B*, IL-2 production was assessed for the selected microproteins using ELISA. Experiments were conducted in triplicates. Data is presented as the mean ± s.d. ∗*p* < 0.05 by Student’s *t* test. A: activated by CD3/CD28. *C* and *D*, validation of the identified microproteins with targeted MS. *Left*, MS/MS mirror spectra of the unique peptide of microproteins. *Right*, relative intensity of the representative microprotein using targeted MS.

To investigate the functional roles of identified microproteins in T cell activation, we selected five up-regulated candidates T1–T5 (Supplemental Table S3). The five microproteins were chosen based on two criteria: (i) the similarity in MS/MS spectra should be above 0.75 (Supplemental Fig. S1); (ii) up-regulation in at least two of three donors under either PMA/Iono or CD3/CD28 activation with fold change above 1.50. Jurkat cells, a widely used T cell line, were stably overexpressed with each microprotein, followed by assessment of IFN-γ and IL-2 production upon CD3/CD28 activation (Fig. 4, *A* and *B*). Compared to activated mock cells, overexpression of T1 and T2 resulted in a significant decrease by 17.1 ∼ 19.0% in both IFN-γ and IL-2 production. Conversely, T3 overexpression led to a noticeable increase by 12.5 ∼ 14.0% in these cytokines. These findings suggest potential regulatory roles for T1-T3 in T cell activation. We further validated the presence of these microproteins in primary T cells using a targeted MS approach, namely parallel reaction monitoring (PRM) (Fig. 4, *C* and *D* and Supplemental Fig. S4). By aligning the experimental MS/MS spectra to theoretical spectra from Prosit database ((https://www.proteomicsdb.org/prosit/), we confirmed the endogenous expression of T1–T3. In addition, PRM was used to quantify T1 and T2 expression in activated primary T cells stimulated by either PMA/Iono or CD3/CD28. Both T1 and T2 were significantly upregulated after activation, regardless of the stimulation method (Fig. 4, *C* and *D*). These results corroborate our proteomic findings and further suggest potential regulatory functions for T1 and T2 during T cell activation.

### Signaling Pathways Related to the Regulatory Function of a Microprotein T1

**Fig. 5:**
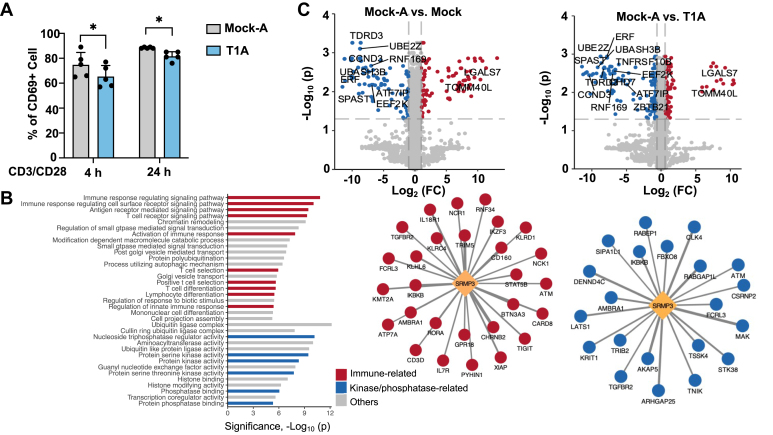
**Signaling pathways and potential mechanisms of T1 as a suppressor of T cell activation**. *A*, flow cytometry analysis of CD69+ in Jurkat cells. Jurkat cells overexpressing T1 or empty vector (Mock) were treated with anti-CD3/CD28 antibodies (4 h or 24 h). Experiments were conducted in triplicates. Data is presented as the mean ± s.d. ∗*p* < 0.05, ∗∗*p* < 0.01 by Student’s *t* test. A: activated by CD3/CD28. *B*, a bioinformatics strategy to predict the function of T1 and its correlated genes in co-expression networks including immune response (*red*) or kinase/phosphatase activity (*blue*). *C*, Volcano plots of the DEPs in T1 overexpressing cells (or Mock cells) during T cell activation. *Red*, proteins with fold change >2 and adjusted *p* value < 0.05; *blue*, proteins with fold change <0.5 and adjusted *p* value < 0.05. A: activated by CD3/CD28. The calculation of adjusted *p* values was performed by Benjamini-Hochberg procedure.

Next, we detected the expression of CD69, a widely accepted marker of activated T cells, to further confirm the regulatory effect of T1 in T cell activation (Fig. 5*A*). Compared to activated wildtype cells, Jurkat cells overexpressing T1 displayed a significant decrease in CD69 expression after CD3/CD28 activation, indicating that overexpressing T1 could inhibit T cell activation and consistent with the results shown in Fig. 4, *A* and *B*. Interestingly, when cells were under short-term activation lasting 4 h, the overexpression of T1 also showed a negative regulation on T cell activation, suggesting T1 may serve as a quick sensor to regulate T cell activation. We examined the transcripts of SRMP3, which contains the sequence of T1. Based on the TCGA RNA-seq data of various tissues, SRMP3 transcript demonstrated significantly higher expression in white blood cells compared to other tissues, aligning with the primary localization of T cells (Supplemental Fig. S5). This observation suggested that T1 was likely to have functions related to immune system. We next applied a bioinformatics strategy to predict the function of T1 (Fig. 5*B*). Over 200 genes positively correlated with the parental gene of T1. The genes were enriched in multiple pathways related to T cell activation and immune response. Moreover, the genes were significantly enriched in pathways related to kinase and phosphatase activity. To further understand the regulatory function of T1, we performed two separate global proteomics experiments to identify DEPs in T cell activation (Fig. 5*C*, Left), as well as DEPs for overexpressed T1 to suppress T cell activation (Fig. 5*C*, Right). We reasoned that the 21 DEPs in common between these two experiments would provide a clue of how T1 suppressed T cell activation as negative feedback. Notably, more than half of these proteins have been reported to be associated with T cell immune pathways. The 13 proteins related to T-cell immunity included SPAST (38), CCND3 (39), UBASH3B (40), UBE2Z (41), TDRD3 (42), ERF (43), TNFRSF10B (44), RNF169 (45), EEF2K (46), CHD7 (47), ATF7IP (48), ZBTB21 (49), and LGALS7 (50), the majority of which showed higher expression in the T1 overexpression group. For example, UBASH3B has been reported to negatively regulate T-cell receptor signaling in activated T-lymphocytes, which may be modulated by T1 and contributed to its immune suppression effect (40).

## Discussion

Inspired by the following considerations, we designed this new combination of proteogenomic method to capture microproteins: (1) microproteins are often characterized by their low abundance and rapid turnover. Given their size and transient nature, microproteins may be synthesized rapidly in response to specific stimuli, making them particularly relevant in dynamic cellular environments. One of the primary strengths of OPP-ID lies in its ability to enrich nascent polypeptides, allowing for the identification of microproteins that are actively being synthesized at the time of analysis (21). We reason that OPP-ID should enhance the detection of low-abundance microproteins that may be overlooked in global proteomic studies, which often prioritize more abundant proteins and rely on longer protein sequences for identification. Therefore, we have proposed involving OPP-ID technology to the new filed of microprotein discovery. (2) While OPP-ID focuses on identifying nascent proteins, it may miss some pre-existing microproteins that are not actively translated during the experimental window. Conversely, global proteomics provides a broader overview of the entire proteome, encompassing both newly synthesized and pre-existing proteins, but may lack the specificity to capture the dynamic changes associated with microprotein synthesis. In this study, we utilized an integrated strategy combining nascent and global proteomics and identified 411 novel microproteins in human T cells, containing 83 nascent and 328 pre-existing microproteins. Interestingly, T2, T3, T4, and T5 are four nascent and upregulated microproteins in activated T cell, but with different regulatory effects on T cell activation. Overexpression of T2 resulted in a significant decrease in both IFN-γ and IL-2 production, T3 overexpression led to a significant increase in these cytokines, while overexpression of T4 and T5 showed almost no effect on T cell activation. The functions of these microproteins remain enigmatic and warrant further investigation.

In addition, our study highlighted the similarities and discrepancies between PMA/Iono and CD3/CD28 activation in both canonical protein and microprotein profiles. For microproteins, 61% differentially expressed microproteins were shared in both distal and proximal signaling pathways, such observation was consistent with canonical proteins. For upregulated pre-existing proteins or nascent microproteins, while most can be triggered by both activation modes, there were specific microproteins that were only upregulated by distal or proximal signaling pathways of T cell activation, presenting an intriguing area for further exploration.

Our results also revealed microprotein T1 may serve as a novel suppressor in the negative feedback loop of T cell activation, which was unknown in immunology. T1 was upregulated during T cell activation, yet its overexpression inhibited this activation. Under conditions of stress, such as during T cell activation, the translation of microproteins like T1 could be significantly altered. This stress-induced translation may lead to the production of microproteins that are not only involved in immediate regulatory functions but also in broader metabolic pathways. The modulation by T1 could serve as a negative feedback mechanism that fine-tunes T cell activation.

In addition, we also noticed there were a few upregulated sORFs that were previously annotated as lncRNA and performed various functions. Interestingly, these lncRNAs were taken granted as noncoding and reported to function at the RNA level, without investigating whether they have translation potential or whether the translated peptides play a role in any biological process. For instance, the microprotein ENST00000663478.1 showed upregulated in T cell activation. The corresponding lncRNA annotated as lnc-SOX6-1 in human bone marrow samples presents high expression in pediatric acute myeloid leukemia (AML) patients, promoting cell proliferation while inhibiting apoptosis (36). Another example is the microprotein IP_724881, which also showed upregulated in T cell activation, the corresponding lncRNA ENST00000566208 has been found as a stable biomarker PBMCs for major depressive disorder (MDD) in individuals, irrespective of gender or age (37). Our study of microproteins from primary T cells have provided a new perspective to look into lncRNA functions and new targets for future studies.

We also recognize that there are several limitations in this study. The combined protein databases without removing redundant protein sequences between databases may increase the risk of false-positive identifications. In future studies, we will consider employing methods to remove redundant sequences or utilize tools specifically designed to address this concern while maintaining the diversity of the protein database. Additionally, when TMT labeling is applied, MS3 approach is ideal for more accurate quantification. Because of compression effect, we likely missed more differentially expressed microproteins. It is also possible that the changes were bigger than what we observed. It could be improved with the MS3 approach and the inclusion of blank channels to reduce artefacts.

## Data availability

The data that support the findings of this study have been deposited to the ProteomeXchange Consortium (http://proteomecentral.proteomexchange.org) *via* the PRIDE partner repository with the dataset identifier PXD058995. Username: reviewer_pxd058995@ebi.ac.uk; Password: seRujnIiDlAg.

## Supplemental data

This article contains supplemental data. (51, 52, 53, 54, 55, 56, 57, 58, 59, 60, 61, 62, 63, 64, 65, 66, 67, 68, https://github.com/najoshi/sickle).

## Conflict of interest

The authors declare that they have no conflicts of interest with the contents of this article.

## References

[1] Wright B.W., Yi Z., Weissman J.S., Chen J. (2022). The dark proteome: translation from noncanonical open reading frames. Trends Cell Biol..

[2] Prensner J.R., Abelin J.G., Kok L.W., Clauser K.R., Mudge J.M., Ruiz-Orera J. (2023). What can Ribo-seq, immunopeptidomics, and proteomics tell us about the noncanonical proteome?. Mol. Cell Proteomics.

[3] Yang Y., Wang H., Zhang Y., Chen L., Chen G., Bao Z. (2023). An optimized proteomics approach reveals novel alternative proteins in mouse liver development. Mol. Cell Proteomics.

[4] Cleyle J., Hardy M.P., Minati R., Courcelles M., Durette C., Lanoix J. (2022). Immunopeptidomic analyses of colorectal cancers with and without microsatellite instability. Mol. Cell Proteomics.

[5] Chen Y., Su H., Zhao J., Na Z., Jiang K., Bacchiocchi A. (2023). Unannotated microprotein EMBOW regulates the interactome and chromatin and mitotic functions of WDR5. Cell Rep..

[6] Nelson B.R., Makarewich C.A., Anderson D.M., Winders B.R., Troupes C.D., Wu F. (2016). A peptide encoded by a transcript annotated as long noncoding RNA enhances SERCA activity in muscle. Science.

[7] Zhang S., Reljić B., Liang C., Kerouanton B., Francisco J.C., Peh J.H. (2020). Mitochondrial peptide BRAWNIN is essential for vertebrate respiratory complex III assembly. Nat. Commun..

[8] Chu Q., Martinez T.F., Novak S.W., Donaldson C.J., Tan D., Vaughan J.M. (2019). Regulation of the ER stress response by a mitochondrial microprotein. Nat. Commun..

[9] Shah K., Al-Haidari A., Sun J., Kazi J.U. (2021). T cell receptor (TCR) signaling in health and disease. Signal. Transduct. Target. Ther..

[10] Sinha P., Clements V.K., Miller S., Ostrand-Rosenberg S. (2005). Tumor immunity: a balancing act between T cell activation, macrophage activation and tumor-induced immune suppression. Cancer Immunol. Immunother..

[11] Hwang J.R., Byeon Y., Kim D., Park S.G. (2020). Recent insights of T cell receptor-mediated signaling pathways for T cell activation and development. Exp. Mol. Med..

[12] Jiao J., Zhao X., Hou R., Wang Y., Chang W., Liang N. (2019). Comparison of two commonly used methods for stimulating T cells. Biotechnol. Lett..

[13] Salerno F., Paolini N.A., Stark R., von Lindern M., Wolkers M.C. (2017). Distinct PKC-mediated posttranscriptional events set cytokine production kinetics in CD8(+) T cells. Proc. Natl. Acad. Sci. U. S. A..

[14] Feske S., Giltnane J., Dolmetsch R., Staudt L.M., Rao A. (2001). Gene regulation mediated by calcium signals in T lymphocytes. Nat. Immunol..

[15] Thaker Y.R., Schneider H., Rudd C.E. (2015). TCR and CD28 activate the transcription factor NF-κB in T-cells *via* distinct adaptor signaling complexes. Immunol. Lett..

[16] Esensten J.H., Helou Y.A., Chopra G., Weiss A., Bluestone J.A. (2016). CD28 costimulation: from mechanism to therapy. Immunity.

[17] Li Y.F., Zheng Y., Vemireddy L.R., Panda S.K., Jose S., Ranjan A. (2018). Comparative transcriptome and translatome analysis in contrasting rice genotypes reveals differential mRNA translation in salt-tolerant Pokkali under salt stress. BMC Genomics.

[18] Tahmasebi S., Khoutorsky A., Mathews M.B., Sonenberg N. (2018). Translation deregulation in human disease. Nat. Rev. Mol. Cell Biol..

[19] Cabrera-Cabrera F., Tull H., Capuana R., Kasvandik S., Timmusk T., Koppel I. (2023). Cell type-specific labeling of newly synthesized proteins by puromycin inactivation. J. Biol. Chem..

[20] Bukau B., Deuerling E., Pfund C., Craig E.A. (2000). Getting newly synthesized proteins into shape. Cell.

[21] Forester C.M., Zhao Q., Phillips N.J., Urisman A., Chalkley R.J., Oses-Prieto J.A. (2018). Revealing nascent proteomics in signaling pathways and cell differentiation. Proc. Natl. Acad. Sci. U. S. A..

[22] Zhang Y.L., Yang Y., Li K.C., Chen L., Yang Y., Yang C.X. (2024). Enhanced discovery of alternative proteins in mouse cardiac development using data-independent acquisition(DIA) proteomics. Anal. Chem..

[23] Matsumoto R., Wang D., Blonska M., Li H., Kobayashi M., Pappu B. (2005). Phosphorylation of CARMA1 plays a critical role in T cell receptor-mediated NF-κB activation. Immunity.

[24] Rade M., Böhlen S., Neuhaus V., Löffler D., Blumert C., Merz M. (2023). A time-resolved meta-analysis of consensus gene expression profiles during human T-cell activation. Genome Biol..

[25] Weerakoon H., Mohamed A., Wong Y., Chen J., Senadheera B., Haigh O. (2024). Integrative temporal multi-omics reveals uncoupling of transcriptome and proteome during human T cell activation. Npj Syst. Biol. Appl..

[26] Hung M.H., Lee J.S., Ma C., Diggs L.P., Heinrich S., Chang C.W. (2021). Tumor methionine metabolism drives T-cell exhaustion in hepatocellular carcinoma. Nat. Commun..

[27] Tomé M., Pappalardo A., Soulet F., López J.J., Olaizola J., Leger Y. (2019). Inactivation of proprotein convertases in T cells inhibits PD-1 expression and creates a favorable immune microenvironment in colorectal cancer. Cancer Res..

[28] Baer A., Colon-Moran W., Bhattarai N. (2018). Characterization of the effects of immunomodulatory drug fingolimod (FTY720) on human T cell receptor signaling pathways. Sci. Rep..

[29] O’Sullivan D., Stanczak M.A., Villa M., Uhl F.M., Corrado M., Klein Geltink R.I. (2021). Fever supports CD8^+^ effector T cell responses by promoting mitochondrial translation. Proc. Natl. Acad. Sci. U. S. A..

[30] Han Q., Bagheri N., Bradshaw E.M., Hafler D.A., Lauffenburger D.A., Love J.C. (2012). Polyfunctional responses by human T cells result from sequential release of cytokines. Proc. Natl. Acad. Sci. U. S. A..

[31] Bevilacqua A., Li Z., Ho P.C. (2022). Metabolic dynamics instruct CD8+ T-cell differentiation and functions. Eur. J. Immunol..

[32] Lee J.H., Lee B.H., Jeong S., Joh C.S., Nam H.J., Choi H.S. (2023). Single-cell RNA sequencing identifies distinct transcriptomic signatures between PMA/ionomycin- and αCD3/αCD28-activated primary human T cells. Genomics Inform..

[33] Rampioni Vinciguerra G.L., Capece M., Scafetta G., Rentsch S., Vecchione A., Lovat F. (2024). Role of Fra-2 in cancer. Cell Death Differ..

[34] Mudge J.M., Ruiz-Orera J., Prensner J.R., Brunet M.A., Calvet F., Jungreis I. (2022). Standardized annotation of translated open reading frames. Nat. Biotechnol..

[35] Zu T., Gibbens B., Doty N.S., Gomes-Pereira M., Huguet A., Stone M.D. (2011). Non-ATG-initiated translation directed by microsatellite expansions. Proc. Natl. Acad. Sci. U. S. A..

[36] Guan X., Wen X., Xiao J., An X., Yu J., Guo Y. (2019). Lnc-SOX6-1 upregulation correlates with poor risk stratification and worse treatment outcomes, and promotes cell proliferation while inhibits apoptosis in pediatric acute myeloid leukemia. Int. J. Lab. Hematol..

[37] Cui X., Sun X., Niu W., Kong L., He M., Zhong A. (2016). Long non-coding RNA: potential diagnostic and therapeutic biomarker for major depressive disorder. Med. Sci. Monit..

[38] Wali G., Siow S.F., Liyanage E., Kumar K.R., Mackay-Sim A., Sue C.M. (2023). Reduced acetylated α-tubulin in SPAST hereditary spastic paraplegia patient PBMCs. Front. Neurosci..

[39] Sicinska E., Aifantis I., Le Cam L., Swat W., Borowski C., Yu Q. (2003). Requirement for cyclin D3 in lymphocyte development and T cell leukemias. Cancer Cell.

[40] Wang Z., Wang Y., Peng M., Yi L. (2019). UBASH3B is a novel prognostic biomarker and correlated with immune infiltrates in prostate cancer. Front. Oncol..

[41] Lee J.Y., An E.K., Hwang J., Jin J.O., Lee P.C.W. (2021). Ubiquitin activating enzyme UBA6 regulates Th1 and Tc1 cell differentiation. Cells.

[42] Deater M., Tamhankar M., Lloyd R.E. (2022). TDRD3 is an antiviral restriction factor that promotes IFN signaling with G3BP1. PLoS Pathog..

[43] Tsiomita S., Liveri E.M., Vardaka P., Vogiatzi A., Skiadaresis A., Saridis G. (2022). ETS2 repressor factor (ERF) is involved in T lymphocyte maturation acting as regulator of thymocyte lineage commitment. J. Leukoc. Biol..

[44] Dufva O., Koski J., Maliniemi P., Ianevski A., Klievink J., Leitner J. (2020). Integrated drug profiling and CRISPR screening identify essential pathways for CAR T-cell cytotoxicity. Blood.

[45] Wang J., Chen H., Deng Q., Chen Y., Wang Z., Yan Z. (2022). High expression of RNF169 is associated with poor prognosis in pancreatic adenocarcinoma by regulating tumour immune infiltration. Front. Genet..

[46] Das J.K., Ren Y., Kumar A., Peng H.Y., Wang L., Xiong X. (2022). Elongation factor-2 kinase is a critical determinant of the fate and antitumor immunity of CD8(+) T cells. Sci. Adv..

[47] Writzl K., Cale C.M., Pierce C.M., Wilson L.C., Hennekam R.C. (2007). Immunological abnormalities in CHARGE syndrome. Eur. J. Med. Genet..

[48] Sin J.H., Kashyap S., Acenas D., Cortez J.T., Lee J., Marson A. (2022). ATF7ip targets transposable elements for H3K9me3 deposition to modify CD8(+) T cell effector and memory responses. J. Immunol..

[49] Sun Y., Preiss N.K., Valenteros K.B., Kamal Y., Usherwood Y.K., Frost H.R. (2020). Zbtb20 restrains CD8 T cell immunometabolism and restricts memory differentiation and antitumor immunity. J. Immunol..

[50] Wu G., Deng W., Chen H.Y., Cho H.J., Kim J. (2024). Galectin 7 leads to a relative reduction in CD4+ T cells, mediated by PD-1. Sci. Rep..

[51] Martin M. (2011). Cutadapt removes adapter sequences from high-throughput sequencing reads. EMBnet J.

[52] Langmead B., Salzberg S.L. (2012). Fast gapped-read alignment with Bowtie 2. Nat. Methods..

[53] Dobin A., Davis C.A., Schlesinger F., Drenkow J., Zaleski C., Jha S. (2013). STAR: ultrafast universal RNA-seq aligner. Bioinformatics.

[54] Zhang P., He D., Xu Y., Hou J., Pan B.F., Wang Y. (2017). Genome-wide identification and differential analysis of translational initiation. Nat. Commun..

[55] Calviello L., Hirsekorn A., Ohler U. (2020). Quantification of translation uncovers the functions of the alternative transcriptome. Nat. Struct. Mol. Biol.

[56] Fields A.P., Rodriguez E.H., Jovanovic M., Stern-Ginossar N., Haas B.J., Mertins P. (2015). A regression-based analysis of ribosome-profiling data reveals a conserved complexity to mammalian translation. Mol. Cell..

[57] Xiao Z., Huang R., Xing X., Chen Y., Deng H., Yang X. (2018). De novo annotation and characterization of the translatome with ribosome profiling data. Nucleic Acids Res..

[58] Raj A., Wang S.H., Shim H., Harpak A., Li Y.I., Engelmann B. (2016). Thousands of novel translated open reading frames in humans inferred by ribosome footprint profiling. elife.

[59] Choudhary S., Li W., Smith A.D. (2020). Accurate detection of short and long active ORFs using Ribo-seq data. Bioinformatics.

[60] Xu Z., Hu L., Shi B., Geng S., Xu L., Wang D. (2018). Ribosome elongating footprints denoised by wavelet transform comprehensively characterize dynamic cellular translation events. Nucleic Acids Res.

[61] Malone B., Atanassov I., Aeschimann F., Li X., Großhans H., Dieterich C. (2017). Bayesian prediction of RNA translation from ribosome profiling. Nucleic Acids Res..

[62] Ji Z. (2018). RibORF: Identifying genome-wide translated open reading frames using ribosome profiling. Curr. Protoc. Mol. Biol..

[63] Erhard F., Halenius A., Zimmermann C., L’Hernault A., Kowalewski D.J., Weekes M.P. (2018). Improved Ribo-seq enables identification of cryptic translation events. Nat. Methods..

[64] Wang H., Wang Y., Yang J., Zhao Q., Tang N., Chen C. (2021). Tissue-and stage-specific landscape of the mouse translatome. Nucleic Acids Res.

[65] Leblanc S., Yala F., Provencher N., Lucier J.-F., Levesque M., Lapointe X. (2024). OpenProt 2.0 builds a path to the functional characterization of alternative proteins. Nucleic Acids Res..

[66] Li Y., Zhou H., Chen X., Zheng Y., Kang Q., Hao D. (2021). SmProt: a reliable repository with comprehensive annotation of small proteins identified from ribosome profiling. Genomics Proteomics Bioinformatics.

[67] Olexiouk V., Crappé J., Verbruggen S., Verhegen K., Martens L., Menschaert G. (2016). sORFs. org: a repository of small ORFs identified by ribosome profiling. Nucleic Acids Res.

[68] Mudge J.M., Ruiz-Orera J., Prensner J.R., Brunet M.A., Calvet F., Jungreis I. (2022). Standardized annotation of translated open reading frames. Nat. Biotechnol..

