# Supplementary material for: Proteogenomics Reveals Microproteins in Activated T Cells
Source: Mol Cell Proteomics. 2025 Feb 4;24(6):100914. doi: 10.1016/j.mcpro.2025.100914 (PMC12289530; doi:10.1016/j.mcpro.2025.100914)
Supplement: Supporting Information [file mmc1.docx]

**Supporting Information**

**Proteogenomics Reveals Microproteins in Activated T Cells**

Yang Yang^1,2^, Chuangmiao Chen^3^, Kecheng Li^1^, Yuanliang Zhang^1^, Lei Chen^1,4^, Jue Shi^5^, Quanhua Mu^1^, Yang Xu^3^, Qian Zhao*^1,2^

1. State Key Laboratory of Chemical Biology and Drug Discovery, Department of Applied Biology and Chemical Technology, The Hong Kong Polytechnic University, Hong Kong, SAR, China

2. Centre for Eye and Vision Research, 17W Hong Kong Science Park, Hong Kong, SAR, China

3. School of Medicine, Southern University of Science and Technology, Shenzhen, China

4. Laboratory for Synthetic Chemistry and Chemical Biology Limited, Hong Kong, China

5. Center and Quantitative Systems Biology, Department of Physics, Hong Kong Baptist University, Hong Kong SAR, China

^*^Corresponding Author, Email: q.zhao@polyu.edu.hk

**Table of Contents**

**I. Supplementary methods.**

1. sORF database construction from RNA-seq and Ribo-seq data
2. Re-annotation of translated open reading frames
3. Co-expression network and function prediction of microprotein

**II. Supplementary results**

**Fig. S1** Correlation plots of the predicted retention time of identified peptides from microproteins in nascent microprotein profiling data. R square was calculated by Pearson method and the MS/MS similarity score (spectral angle, SA) was indicated by colored dots. The closer the similarity score approaches 1.0, the greater the similarity between the experimental spectrum and the theoretical spectrum, indicating a more reliable identification.

**Fig. S2** Venn-diagrams of the DEGs and DEPs that were identified using (A) RNA-seq and (B) proteomics approach in each activation group.

**Fig. S3** A PCA plot of three doners in proteomics results.

**Fig. S4** Typical MS/MS spectra and targeted MS results of the detected peptide of T3 (IP_686508), T4 (IP_584493), and T5 (IP_574710) in primary T cells.

**Fig. S5** The expression distribution of gene SRMP3 in human organs based on TCGA database.

**III. Supplementary table**

Table 1 Protein list of global proteome landscape of T-cell activation triggered by PMA/Iono and CD3/CD28 stimulation.

Table 2 Abundance of unique peptide sequence of microproteins in nascent proteome under T-cell activation triggered by PMA/Iono (PI) and CD3/CD28 (CD) stimulation.

Table 3 Unique peptide and fold changes in T cell activation of microprotein T1, T2, T3, T4 and T5.

**I. Supplementary methods**

**sORF database construction from RNA-seq and Ribo-seq data**

A homemade bioinformatic pipeline was used to generate ORFs database using RNAseq data. Briefly, Raw reads of RNAseq were firstly clean up by fastp (v0.23.2). 5' Adapter sequence (AGATCGGAAGAGCGTCGTGTAGGGAAAGAGTGTAGATCTCGGTGGTCGCCGTATCATT) and 3' adapter sequence (GATCGGAAGAGCACACGTCTGAACTCCAGTCACGGATGACTATCTCGTATGCCGTCTTCTGCTTG) were used to trim retained adapter sequence. Next, clean reads were aligned to human genome (GRCh38.p13) by hisat2(v2.2.1) with default parameter. All possible transcripts were assembled using stringtie (v2.2.0). Then, read count of transcripts were calculated by featureCounts(v2.0.3). Transcripts with read count in more than half samples were filtered out for three-frame translation. Biopython toolkit was applied to translated transcript sequences into protein sequences with AUG as start codon.

Next, a comprehensive ORF database with translational evidence was constructed by using 172 public Riboseq datasets. Briefly, preprocessing of Ribo-seq raw data included adaptor removal using Cutadapt(51) (v 2.4, with parameters “--minimum-length 6 --discard-untrimmed --match-read-wildcards --max-n = 0.5”), low-quality trimming using Sickle(52) (v 1.33, with parameters “se -x -t sanger”). rRNA and tRNA contaminants were removed by aligning trimmed reads to human tRNA and rRNA sequences using Bowtie 2(53) (v1.0.1, with command “-q -L 20 --phred33 --end-to-end”). All remaining reads were mapped to the human reference genome GRCh38.p14 with a GTF annotation file (GENCODE v44) using STAR(54) (v 2.7.2 a) , and further unique mapped reads were extracted. Ten pipelines, RiboTISH(55) (v 0.2.1), ORFquant(56) (v 0.99.0), ORFRATER(57), RiboCode(58) (v 1.2.11), riboHMM(59), Ribotricer(60) (v 1.3.1), RiboWave(61) (v 1.0), RP-BP(62) (v 2.0.0), RibORF(63) (v 1.0), and PRICE(64) (v 1.0.3 b), were used to perform ORF detection with the longest strategy under the default threshold setting. The final set of actively translated ORFs with all near-cognate start codons (AUG, TUG, CUG, and GUG) followed by an in-frame stop codon in annotated transcripts was stringently filtered based on the requirement of a minimum length of 18 nucleotides and the expression of the ORF-containing gene at an above-background level, as described in a previous report(65).

**Re-annotation of translated open reading frames**

Our sORFs were identified from different resource and databases, including OpenProt(66), SmProt(67), sORFs.org(68), RNAseq three-frame translation and Riboseq prediction. To unify their genomic annotation, we adopted an annotation framework proposed by scientific community(69). First, all genomic coordinates of sORFs were unified to human genome version GRCh38.p13. Next, their coordinates were overlapped with reference transcripts from Ensembl and NCBI database. Overlapped transcripts were translated into protein sequence to match against amino acid sequence of sORF candidates. The sORFs were categorized into several different group based on their relative location to nearest ORF in the transcript.

**Co-expression network and function prediction of microprotein**

A computational method is designed to predict the function of the microprotein based on gene co-expression networks. We first examined the transcriptomic expression of the parental gene of T1, SRMP3, in different normal tissues in public databases, including Genotype-Tissue Expression (GTEx) and The Cancer Genome Atlas (TCGA). Since white blood cells had the highest SRMP3 expression, we constructed a gene co-expression network in white blood cell samples (n = 173) based on Spearman’s correlation coefficient. The genes were then ranked by their correlation coefficient. Gene set enrichment analysis was then performed based on the ranking using the fgsea package. We focused on Gene Ontology (GO) pathways obtained from the Molecular Signature Database (https://www.gsea-msigdb.org/gsea/msigdb/human/genesets.jsp?collection=C5), including those belonging to biological processes (BP), cellular components (CC), and molecular functions (MF). The pathways with names containing “immune,” “immunity,” “T cell,” “antigen,” or “lymphocyte” were considered immune-related, and pathways containing “kinase” and “phosphatase” were considered kinase/phosphatase related. The member genes of these pathways with a correlation coefficient higher than 0.35 were visualized in the networks by Cytoscape.

References:

51. M. Martin, Cutadapt removes adapter sequences from high-throughput sequencing reads, EMBnet. journal, 17 (1), 2011, 10–12.

52. N. A. Joshi and J. N. Fass, Sickle: a sliding-window, adaptive, quality-based trimming tool for FastQ files, 2011, https://github.com/najoshi/sickle.

53. B. Langmead and S. L. Salzberg, Fast gapped-read alignment with Bowtie 2, Nat. Methods., 9 (4), 2012, 357–359.

54. A. Dobin, C. A. Davis, F. Schlesinger, J. Drenkow, C. Zaleski, et al., STAR: ultrafast universal RNA-seq aligner, Bioinformatics, 29 (1), 2013, 15–21.

55. P. Zhang, D. He, Y. Xu, J. Hou, B. F. Pan, et al., Genome-wide identification and differential analysis of translational initiation, Nat. Commun., 8 (1), 2017, 1749.

56. L. Calviello, A. Hirsekorn and U. Ohler, Quantification of translation uncovers the functions of the alternative transcriptome, Nat. Struct. Mol. Biol, 27 (8), 2020, 717–725.

57. A. P. Fields, E. H. Rodriguez, M. Jovanovic, N. Stern-Ginossar, B. J. Haas, et al., A regression-based analysis of ribosome-profiling data reveals a conserved complexity to mammalian translation, Mol. Cell, 60 (5), 2015, 816–827.

58. Z. Xiao, R. Huang, X. Xing, Y. Chen, H. Deng and X. Yang, De novo annotation and characterization of the translatome with ribosome profiling data, Nucleic Acids Res., 46 (10), 2018, e61–e61.

59. A. Raj, S. H. Wang, H. Shim, A. Harpak, Y. I. Li, et al., Thousands of novel translated open reading frames in humans inferred by ribosome footprint profiling, elife, 5, 2016, e13328.

60. S. Choudhary, W. Li and A. D. Smith, Accurate detection of short and long active ORFs using Ribo-seq data. Bioinformatics, 36(7), 2020, 2053-2059.

61. Z. Xu, L. Hu, B. Shi, S. Geng, L. Xu, et al., Ribosome elongating footprints denoised by wavelet transform comprehensively characterize dynamic cellular translation events, Nucleic Acids Res., 46 (18), 2018, e109-e109.

62. B. Malone, I. Atanassov, F. Aeschimann, X. Li, H. Großhans and C. Dieterich, Bayesian prediction of RNA translation from ribosome profiling, Nucleic Acids Res., 45 (6), 2017, 2960–2972.

63. Ji, RibORF: Identifying genome‐wide translated open reading frames using ribosome profiling. Curr. Protoc. Mol. Biol., 124 (1), 2018, e67.

64. F. Erhard, A. Halenius, C. Zimmermann, A. L’Hernault, D. J. Kowalewski, et al., Improved Ribo-seq enables identification of cryptic translation events, Nat. Methods, 15 (5), 2018, 363–366.

65. H. Wang, Y. Wang, J. Yang, Q. Zhao, N. Tang, et al., Tissue-and stage-specific landscape of the mouse translatome, Nucleic Acids Res., 49 (11), 2021, 6165–6180.

66. S. Leblanc, F. Yala, N. Provencher, J.-F. Lucier, M. Levesque, et al., OpenProt 2.0 builds a path to the functional characterization of alternative proteins. Nucleic Acids Res., 52(D1), 2024, D522-D528.

67. Y. Li, H. Zhou, X. Chen, Y. Zheng, Q. Kang, et al., SmProt: a reliable repository with comprehensive annotation of small proteins identified from ribosome profiling, Genomics, proteomics & bioinformatics, 19 (4), 2021, 602–610.

68. V. Olexiouk, J. Crappé, S. Verbruggen, K. Verhegen, L. Martens and G. Menschaert, sORFs. org: a repository of small ORFs identified by ribosome profiling. Nucleic Acids Res., 44(D1), 2016, D324-D329.

69. J. M. Mudge, J. Ruiz-Orera, J. R. Prensner, M. A. Brunet, F. Calvet, et al., Standardized annotation of translated open reading frames, Nat. Biotechnol., 40 (7), 2022, 994–999.

**II. Supplementary results**


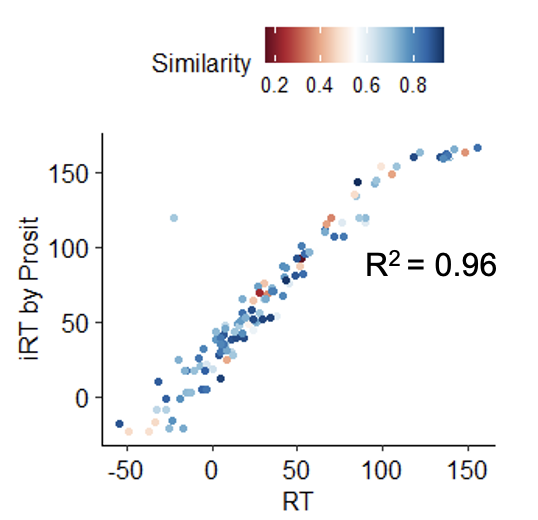


**Fig. S1** Correlation plots of the predicted retention time of identified peptides from microproteins in nascent microprotein profiling data. R square was calculated by Pearson method and the MS/MS similarity score (spectral angle, SA) was indicated by colored dots. The closer the similarity score approaches 1.0, the greater the similarity between the experimental spectrum and the theoretical spectrum, indicating a more reliable identification.

**
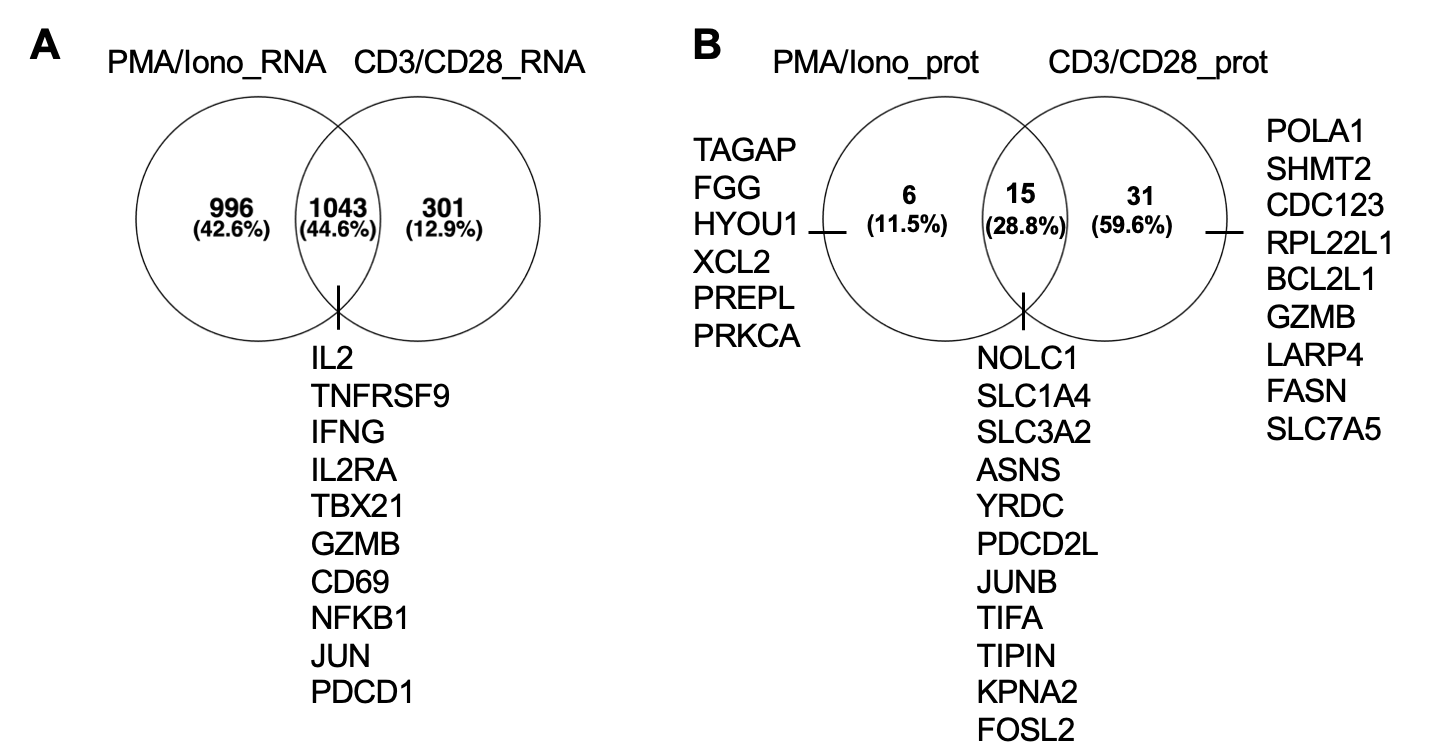
**

**Fig. S2** Venn-diagrams of the differentially expressed genes (DEGs) and proteins (DEPs) that were identified using (A) RNA-seq and (B) proteomics approach in each activation group.


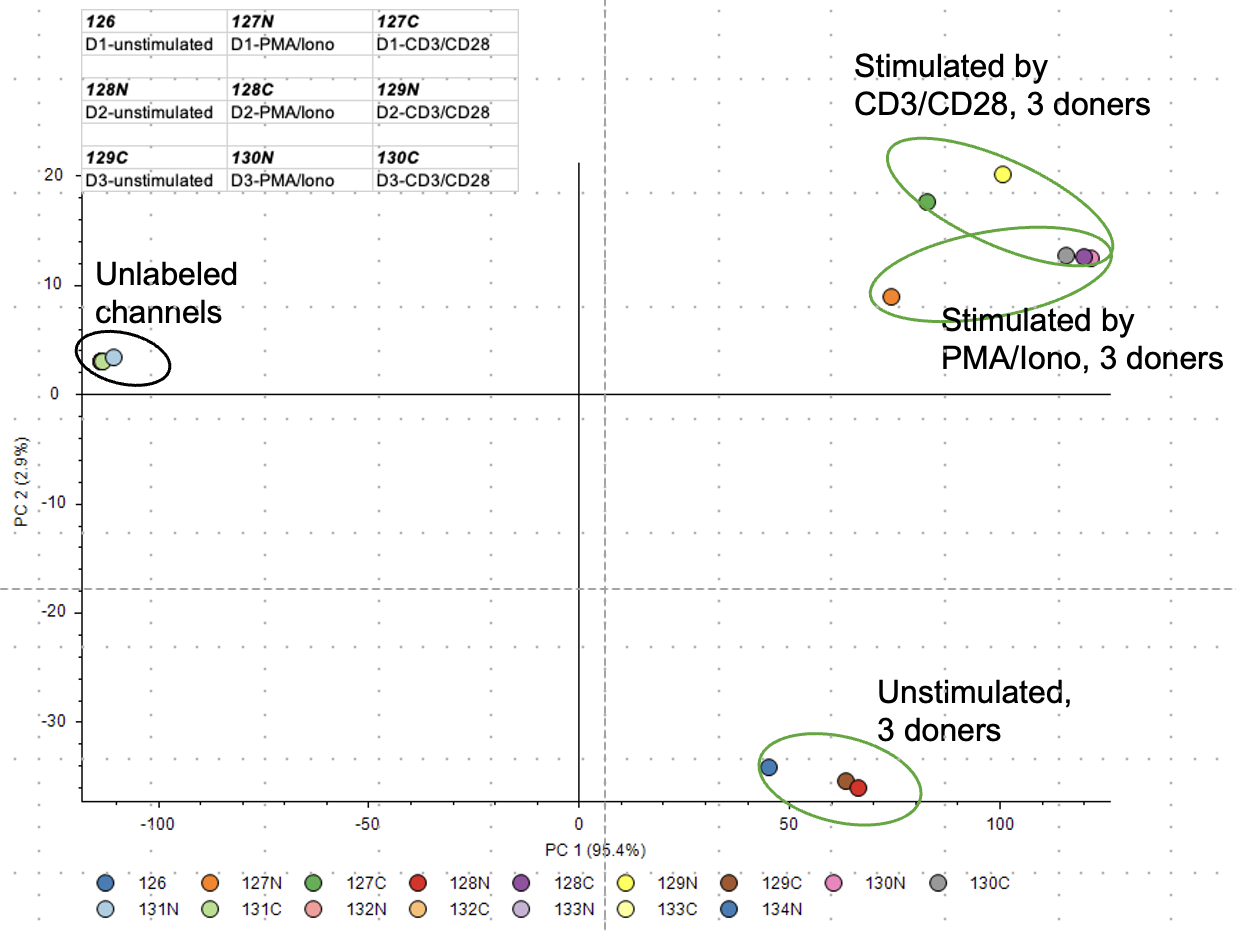


**Fig. S3** A PCA plot of three doners in proteomics results.


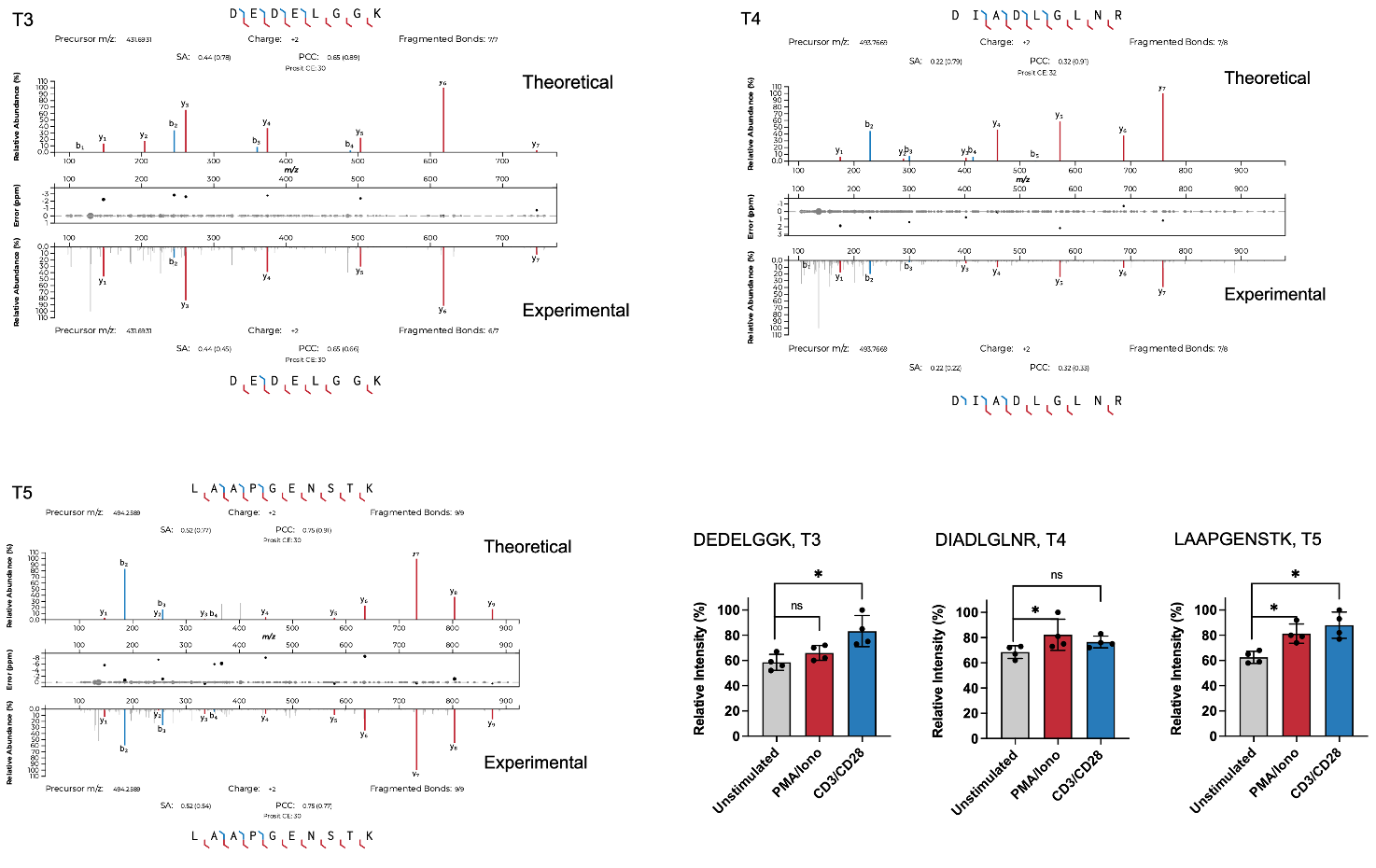


**Fig. S4** Typical MS/MS spectra and targeted MS results of the detected peptide of T3 (IP_686508), T4 (IP_584493), and T5 (IP_574710) in primary T cells.


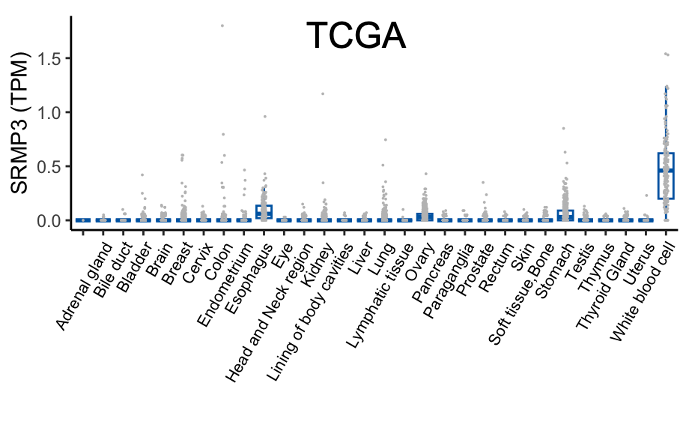


**Fig. S5** The expression distribution of gene SRMP3 in human organs based on TCGA database.
